# C3 Glomerulopathy: recent advances and an update on management

**DOI:** 10.1590/2175-8239-JBN-2025-0306en

**Published:** 2026-06-12

**Authors:** Lilian Monteiro Pereira Palma, Maria Izabel Neves de Holanda Barbosa, Sanjeev Sethi

**Affiliations:** 1 Universidade Estadual de Campinas, Faculdade de Ciências Médicas, Nefrologia Pediátrica, Campinas, SP, Brazil.; 2 Hospital Federal de Bonsucesso, Nefrologia, Rio de Janeiro, RJ, Brazil.; 3 Mayo Clinic, Department of Laboratory Medicine and Pathology, Rochester, MN, United States.

**Keywords:** Glomerulonephritis, Complement C3, Glomerulonephritis, Membranoproliferative

## Abstract

C3 glomerulopathy (C3G) is a clinicopathologic entity characterized by glomerular inflammation with dominant staining for C3 on immunofluorescence microscopy. Electron microscopy is used to determine the location and type of deposits, which further divides C3G into C3 glomerulonephritis (C3GN) and dense deposit disease (DDD) based on ultrastructural findings. Both entities are progressive, with about 60% of patients progressing to end-stage kidney disease (ESKD) within 10 years. Recurrence after kidney transplantation occurs in up to 60% of C3G cases. New knowledge about the pathophysiology—different patterns of injury, histopathological scoring, apolipoprotein E staining, dysregulation of the alternative complement pathway (including both autoantibodies to and mutations in complement regulatory factors), as well as proteomic analyses—has allowed a better understanding of different pathophysiological profiles and clinical presentations, enabling individualized treatment. The role of nephroprotection is well established in C3G, as it is for other glomerular diseases. Non-specific treatment with corticosteroids and immunosuppression is associated with a variable response rate. However, more recently, results from clinical trials with proximal complement inhibitors have shown significant improvement in proteinuria and attenuation of the decline in estimated glomerular filtration rate (eGFR). Based on the results of these trials involving drugs that specifically target the upstream complement pathways, including the alternative pathway, new treatment options have recently become available for patients with C3G.

## Introduction

**Figure 1:**
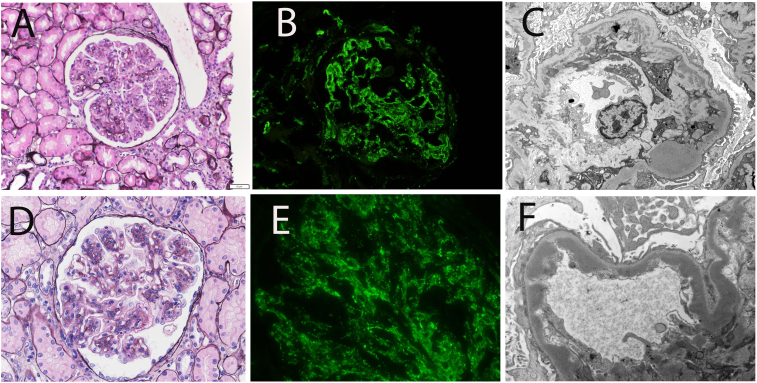
Biopsy findings of C3 glomerulopathy (C3G). C3G is characterized by bright glomerular staining for C3 on immunofluorescence microscopy (IF), in the absence of significant immunoglobulin (Ig) deposits. By definition, the intensity of C3 is at least two orders of magnitude greater than that of any Ig. Conversely, when there is bright Ig staining on IF, with or without C3, the entity is called immune complex-mediated glomerulonephritis (IC-MPGN). C3G is further subdivided into C3GN and dense deposit disease (DDD) based on electron microscopy (EM). In C3GN, the deposits are subendothelial, subepithelial, or sparse intramembranous, while in DDD, the deposits are sausage-shaped, dense (osmiophilic), and intramembranous. The top panel shows the biopsy findings in a case of C3GN, and the bottom panel shows those of DDD. Both C3GN and DDD show an MPGN pattern of injury (A and D), bright C3 (B and E), with absence of Ig (not shown), numerous subendothelial deposits in C3GN (C), and numerous dense, osmiophilic, intramembranous deposits in DDD (F).

C3 glomerulopathy (C3G) is a clinicopathologic entity (i.e., it requires a kidney biopsy for diagnosis), usually (but not exclusively) characterized by a membranoproliferative pattern (MPGN) of injury on light microscopy, with a predominance of C3 staining on immunofluorescence microscopy. C3G is further subdivided into C3 glomerulonephritis (C3GN) and dense deposit disease (DDD) based on electron microscopy (EM) (Figure 1). Factors that dysregulate the alternative complement pathway (genetic variants and/or autoantibodies) are present in a variable percentage of patients. Serum C3 levels are decreased in approximately 60% of cases. Progression to end-stage kidney disease (ESKD) within 10 years is 70% in children and 30%–50% in adults^1^. In addition, patients with DDD reportedly progress at twice the rate of C3GN patients^2,3^. There is also a high rate of recurrence after kidney transplantation (up to 89% in a case series) at a median (interquartile range) of 33 (13-141) days after transplantation^4^. While short-term graft survival in cases with recurrence is favorable, long-term graft loss may reach 50% at a median time of 77 months^5^.

The membranoproliferative glomerulonephritis (MPGN) was previously classified into types I, II, and III based on the location of electron-dense deposits by EM^6,7,8^. This classification did not consider the underlying etiology of the MPGN pattern of injury. Thus, the classification was problematic, as different causes of the MPGN pattern were often grouped into a single entity, hindering treatment and the analysis of clinical trial outcomes.

In 2011, Sethi and Fervenza revisited the classification of membranoproliferative glo­merulonephritis (MPGN), proposing that immuno­fluorescence microscopy be the main diagnostic tool to differentiate MPGN mediated by immune complexes/immunoglobulins (Ig) and/or complement deposition^9,10^. The classification of MPGN into immune complex-mediated (IC-MPGN) and complement-mediated MPGN changed the fundamental approach from a morphologic basis to an etiologic basis of the MPGN pattern^11,12^. Thus, immune complex/Ig-mediated MPGN is associated with infections, autoimmune diseases, or paraproteinemia, whereas complement-mediated MPGN is associated with abnormalities of the alternative pathway of complement (Chart 1).

**Chart 1:** Investigation of patients with C3 glomerulonephritis (C3GN) and immune complex-mediated membranoproliferative glomerulonephrits (IC-MPGN)

Rule out secondary causes and evaluate defects in the alternative complement pathway
Secondary causes: • Hepatitis B • Hepatitis C • Human immunodeficiency virus • Protozoal tests • Tropical diseases: malaria, schistosomiasis • Autoimmune diseases • Monoclonal gammopathy (at any age, especially in patients > 50 years of age) • Sickle cell disease • Acute Infections, endocarditis • Streptococcal infections • Malignancies
Quantification of complement factors and regulators: • C3 • C4 • CH50 • AH50 • Factor H, Factor I, Factor B • Anti-factor H antibody • C3 nephritic factor (C3NeF) • C4 nephritic factor (C4NeF) • C5 nephritic factor (C5NeF)
Genetic panel or full EXOME sequencing

At the same time, the term C3G was suggested in 2010 by a French group after analyzing biopsies showing glomerulonephritis with dominant C3 staining on immunofluorescence microscopy, highlighting the possibility of defects in the alternative complement pathway^13,14^. These studies led to a consensus meeting in 2013 to establish a uniform definition of complement-mediated glomerulonephritis, as it was recognized that, although MPGN was the most common pattern of injury, other patterns, including mesangial proliferative, crescentic, and diffuse endocapillary proliferative GN, may also be present in this group of diseases^15^. The terminology “complement-mediated MPGN” was thus replaced by “C3G.”

The importance of correctly classifying MPGN into IC-MPGN and C3G cannot be overstated. Detailed clinical, pathologic, and laboratory evaluation of IC-MPGN often leads to the identification of the underlying etiology, which is then amenable to targeted treatment (Sethi S, Kidney Int, 2026, minor revision submitted). The term primary/idiopathic MPGN is used when an underlying etiology of the immune complexes/Ig is not found. Conversely, the diagnosis of C3G should lead to an evaluation of complement abnormalities, especially in the alternative pathway. These patients are likely to benefit the most from drugs that block complement pathways. Since IC-MPGN is also associated with complement activation and glomerular deposition of these factors, it is easy to envision that blocking this system may also have a beneficial effect in this group of patients.

## Discussion

C3G is an ultra-rare glomerular disease, with an incidence of 1–2 new cases per million population per year, whose most frequent clinical manifestations are hematuria and proteinuria. It may present as nephritic and/or nephrotic syndrome, in some cases, with low C3 levels^16,17^. The Brazilian Society of Nephrology has launched the C3G/IC-MPGN Registry through its Committee of Rare Diseases (comdora-sbn.org.br – restricted access) as there are no data on the prevalence of this disease in the country or region. In addition to kidney involvement, patients with DDD may also present extrarenal manifestations, such as drusen and acquired lipodystrophy^18^.

The pathophysiological basis of C3G is dysregulation of the alternative complement pathway, which can occur due to mutations in complement regulatory proteins (less than 20% of cases) or due to acquired causes. These include C3, C4, and C5 nephritic factors (C3NeF, C4NeF, C5NeF—autoantibodies that stabilize the alternative pathway C3 or C5 convertase), as well as anti-factor B, anti-factor I, and anti-factor H autoantibodies^19^. All these mechanisms lead to excessive complement activation with ensuing glomerular accumulation of complement factors. Given that these factors are potent chemotactic agents, leukocyte influx (proliferative phase) occurs, followed by the formation of a new basement membrane (repair phase), leading to a double-contour appearance on light microscopy, which is the hallmark of the MPGN pattern of injury. Other patterns of injury, in addition to MPGN, may be present, including mesangial proliferative, crescentic, and sclerosing patterns^20,21^. Iatropoulos et al. evaluated 173 patients with C3G and IC-MPGN, separating them into four clusters according to biopsy criteria, serum complement markers, and genetic findings, suggesting variability in treatment response and outcomes among the different groups^21^. Overlapping of C3G and atypical hemolytic uremic syndrome (aHUS) has been described in patients who are more likely to present genetic variants in alternative complement pathway genes rather than nephritic factors^22^.

Most cases of post-infectious glomerulonephritis (PIGN) exhibit an exudative pattern of injury with both granular IgG and bright C3 along the capillary walls on immunofluorescence microscopy. However, it is not uncommon for PIGN to show bright C3 deposition with sparse immunoglobulin on immunofluorescence microscopy. In these cases, the distinction between PIGN and C3GN will depend on the absence of atypical features on light and electron microscopy, as well as a clinical course typical of PIGN, with complete resolution and normalization of serum C3 within eight weeks. However, it is important to note that C3GN may manifest after an infectious episode, and there may be subepithelial humps in C3GN as well as in PIGN^23^. Therefore, the presence of any atypical clinical or histological features in a case of PIGN should raise suspicion for C3GN.

Another significant differential diagnosis is the presence of a monoclonal immunoglobulin, which has been reported in C3GN without clonal deposits in renal tissue^24^. The monoclonal Ig likely acts as an autoantibody to complement regulatory proteins, resulting in dysregulation of the alternative pathway. Therefore, it is recommended that paraproteinemia be investigated in all patients with C3GN (especially those over 50 years of age). Consultation with a hematologist is also recommended, because treating the underlying paraproteinemia with clone-directed therapy often leads to remission of kidney disease in monoclonal Ig-associated C3GN^25^. It should be noted that there is a high rate of recurrence after transplantation in these cases^26^. Thus, the identification of this subgroup of patients becomes even more important.

More recently, laser microdissection and mass spectrometry (LMD/MS) in glomerular diseases have contributed to the understanding of the pathophysiological mechanisms of C3G. The most relevant finding stems from a study led by the Mayo Clinic in Rochester, in which LMD/MS revealed marked accumulation of complement proteins and complement regulatory proteins in both C3GN and DDD compared with controls^27,28^. A six- to nine-fold increase in C5-9 was detected in DDD cases compared with C3GN. Furthermore, a nine-fold increase in the amount of apolipoprotein E (ApoE) was surprisingly found in DDD biopsies compared with C3GN. Validation studies confirmed the diagnosis of C3GN and DDD in 80.6% of cases based solely on ApoE staining. This marker can be used as an adjunct to EM for the diagnosis of DDD and may be valuable when EM is not available^28^.

**Figure 2:**
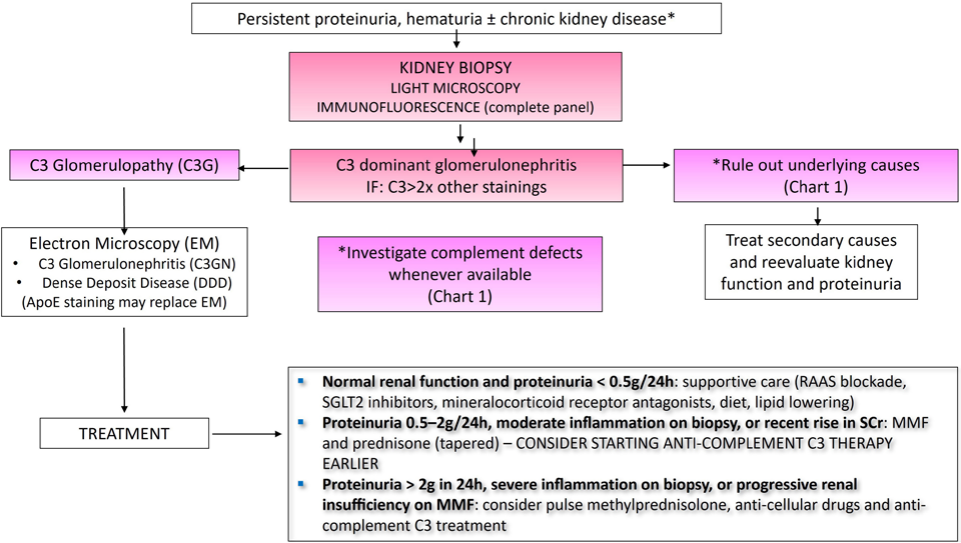
A new proposal for the management of patients with C3 glomerulopathy.

Published in Kidney International in 2021, the Kidney Disease: Improving Global Outcomes (KDIGO) Clinical Practice Guideline for Glomerular Diseases [Kidney Disease: Improving Global Outcomes Glomerular Diseases Work Group]^25^ stratifies the management of C3G and IC-MPGN. The first step (Figure 2) is to treat the underlying condition whenever it is identified (infection, dysproteinemia, autoimmune disease, or others). The second step is to treat patients according to kidney function and the degree of proteinuria. For patients with C3G, normal kidney function, and nephrotic-range proteinuria or active urinary sediment, corticosteroids may be used for 12–16 weeks (prednisone 1 mg/kg/day or equivalent) in conjunction with traditional nephroprotective measures (angiotensin-converting enzyme inhibitors, angiotensin receptor blockers in maximally tolerated doses, diet, lipid control). As glomerular filtration decreases or proteinuria persists, the use of immunosuppressants (especially mycophenolic acid) has been indicated in moderate disease (proteinuria over 500 mg/day, moderate inflammation on biopsy, or an acute rise in serum creatinine)^29^. In the European population, remission with mycophenolic acid was achieved mainly in young patients with C3NeF and autoantibodies. There was a recurrence of proteinuria in 33% of patients who discontinued mycophenolic acid. It is important, however, to highlight that the use of mycophenolic acid in this setting is still off-label, and these results were not reproduced in a case series from the United States^30^. In severe disease, with proteinuria greater than 2 g/day (or severe inflammation on biopsy or progressive loss of kidney function), despite immunosuppression and supportive therapy, methylprednisolone pulse therapy or anti-cellular agents may be used, although efficacy has not been proven.

In cases where there is a crescentic form on biopsy, the combination of cyclophosphamide and high-dose corticosteroids is indicated^25^.

The use of SGLT2i (sodium-glucose cotransporter 2 inhibitors) and mineralocorticoid receptor antagonists has been associated with increased patient and kidney survival, even in non-diabetic patients with an estimated glomerular filtration rate (eGFR) between 25 and 75 mL/min/1.73 m^2^. These therapies are emerging as a non-specific treatment that may be used in patients with C3G and proteinuria^25,31^.

For several years, the only complement inhibitor available on the market was eculizumab, a humanized anti-C5 monoclonal antibody. Off-label use for some cases of C3G has not proven effective in all patients^32,33^, including the European cohort of 97 patients with C3G, of whom nine received eculizumab without benefit for kidney survival^29^. This is most likely due to the downstream effect of eculizumab, whereas the complement defect in C3G is primarily upstream. These findings were demonstrated in a study that evaluated biomarkers in patients with aHUS, C3G, and acute antibody-mediated rejection treated with eculizumab, in which individuals with C3G maintained elevated levels of C3d and consistently low levels of C3 with terminal complement inhibition, reflecting continuous proximal complement activation despite the use of a terminal complement inhibitor^34^.

Recently, novel medications have been approved by the FDA (Food and Drug Administration) for the treatment of C3G^35,36^ (Chart 2).

**Chart 2:** Clinical trials of C3 inhibitors – demographic data and results

Clinical Trial	APPEAR (NCT04817618) – Novartis Pharma	VALIANT (NCT05067127) – Apellis Pharma
Drug	Iptacopan (Factor B inhibitor)	Pegcetacoplan (C3/C3b inhibitor)
Administration	200 mg PO BID	1080 mg SC twice weekly
Disease	C3G, native kidneys (biopsy confirmed within 12 months of enrollment)	C3G (n = 51), IC-MPGN (n = 12) in native kidneys and post-transplant
Study Design	Multicenter, randomized, double-blind, placebo-controlled, phase 3 trial	Multicenter, randomized, double-blind, placebo-controlled, phase 3 trial
Inclusion Criteria	*Adults 18–30 years *Reduced serum C3 concentration (<77 mg/dL [defined as <0.85 × the lower limit of the central laboratory normal range]) at screening *MPA, corticosteroids (prednisone <7.5 mg/day), SGLT2i, and MRA allowed when stable for at least 90 days before randomization *Maximally tolerated doses of angiotensin-converting enzyme inhibitors or AT1-receptor blockers for at least 90 days *uPCR ≥1.0 g/g at day –75 and day –15 before randomization *eGFR ≥30 mL/min/1.73 m^2^ *Vaccination against *Neisseria meningitidis* and *Streptococcus pneumoniae*	*Adolescents (12–17 years) and adults (≥18 years) *Native kidneys and post-kidney transplant recurrence *MPA and corticosteroids (prednisone ≤ 20 mg/day) permitted *Stable, optimized antiproteinuric regimens: ACEi, ARB, SGLT2i *uPCR ≥1.0 g/g in 2 or more first-morning spot urine samples and during screening *eGFR ≥30 mL/min/1.73 m^2^ *Vaccination against *Streptococcus pneumoniae*, *Neisseria meningitidis*, and *Haemophilus influenzae* type B
Exclusion Criteria	*Recipient of any cell or solid organ transplantation (for phase 3) *Rapidly progressive crescentic glomerulonephritis *Kidney biopsy showing interstitial fibrosis or tubular atrophy *Monoclonal gammopathy of undetermined significance	*Evidence of transplant rejection *Diagnosis of secondary C3G or IC-MPGN *Severe infection within 14 days prior to the first dose; recurrent or chronic severe infections or history of meningococcal disease *Evidence of improving kidney disease
Primary Endpoint	Reduction in proteinuria (measured as the log-transformed ratio to baseline of uPCR obtained from a 24-h urine collection) at 6 months	Log-transformed ratio of uPCR at week 26 compared with baseline
Participants (n)	Iptacopan group (38)/placebo group (36)	Pegcetacoplan group (63)—C3G (51), IC-MPGN (12); adults (35); adolescents (28); native kidneys (58), kidney transplantation (5) Placebo group (61)—C3G (45), IC-MPGN (16); adults (34); adolescents (27); native kidneys (57), kidney transplantation (4)
Post-kidney Transplant	Analyzed in a phase 2 trial (11 C3G)	Pegcetacoplan group (5)/placebo group (4)
Pathology	Mean change in total activity score from baseline to 6 months: -1.95 (iptacopan) vs. 1.11 (placebo) (p = 0.2895) Mean change in C3 deposit score from baseline to 6 months: -0.78 (iptacopan) vs. 1.09 (placebo)	Mean change in total activity score from baseline to 6 months: -3.5 (pegcetacoplan) vs. -2.4 (placebo) (p = 0.2753) >2 orders of magnitude reduction in C3c staining 74.3% (pegcetacoplan) vs. 11.8% (placebo)
uPCR change vs. placebo	35.1%	68.3%
Composite kidney endpoint (≥50% reduction in uPCR AND ≤15% reduction in eGFR): week 26 vs. baseline	7.15-fold (95% CI 1.43–35.72) increase in the odds of achieving the composite kidney endpoint compared with placebo (p = 0.0166) Increased eGFR at 6 months: +1.30 mL/min/1.73 m^2^ (iptacopan) vs. -0.86 mL/min/1.73 m^2^ (placebo)	29.4-fold (95% CI 6.5–132.2) higher odds of achieving composite kidney endpoints vs. placebo (p < 0.0001)
Patients with baseline proteinuria >3g/24h (%)	Iptacopan group (71%)/placebo group (58%)	Pegcetacoplan group (38%)/placebo group (23%)
Baseline eGFR (mean)	Iptacopan group (89.3 mL/min/1.73 m^2^)/placebo group (99.2 mL/min/1.73 m^2^)	Mean change in eGFR from baseline: -1.6 mL/min/1.73 m^2^ (pegcetacoplan) vs. -7.9 mL/min/1.73 m^2^ (placebo)
Serious Adverse Events	Iptacopan group (8%)/placebo group (3%)	Pegcetacoplan group (9.5%)—1 death due to COVID-19/placebo group (9.8%)

Abbreviations – uPCR: urinary protein-to-creatinine ratio; eGFR: estimated glomerular filtration rate; C3G: C3 glomerulopathy; IC-MPGN: immune complex-mediated membranoproliferative glomerulonephritis; ACEi: angiotensin-converting enzyme inhibitors; ARB: angiotensin receptor blockers; C3GN: C3 glomerulonephritis; DDD: dense deposit disease; MPA: mycophenolic acid; SGLT2i: sodium-glucose cotransporter 2 inhibitors; MRA: mineralocorticoid receptor antagonists; CI: confidence interval.

The APPEAR-C3G study (Novartis Pharma) was a phase 3, prospective, multicenter, randomized, double-blind, placebo-controlled trial that evaluated the use of iptacopan in patients with C3G—a molecule of the indole class and a potent inhibitor of complement factor B that prevents the unregulated amplification of the C3 convertase. Both arms received supportive care. Iptacopan was administered at a dose of 200 mg orally twice daily. The endpoints included reduction in proteinuria, measured as the urinary protein-to-creatinine ratio (uPCR), and the slope of the eGFR. In patients who completed six months of therapy, iptacopan reduced proteinuria by 35.1% compared with placebo^37^, and 43.5% of patients achieved the composite endpoint (>50% reduction in uPCR + <15% reduction in eGFR) at 12 months.

The VALIANT study (Apellis Pharma), a prospective, randomized, double-blind, phase 3 trial, demonstrated that the use of pegcetacoplan—a pegylated peptide that selectively binds to C3 and prevents its cleavage into C3a and C3b—in patients with C3G and IC-MPGN, compared with placebo for 26 weeks, reduced proteinuria in treated patients by 68.3%. Pegcetacoplan was administered subcutaneously at a dose of 1080 mg twice weekly. The proportion of patients who achieved a composite kidney endpoint (≥50% reduction in the uPCR plus ≤15% reduction in eGFR) at week 26 vs. baseline was 49.2% in the pegcetacoplan group, and there was a 29.4-fold higher odds of achieving composite kidney endpoints vs. placebo (p < 0.0001). In the pegcetacoplan group, there was a gain of 6.3 mL/min/1.73 m^2^ in eGFR compared with placebo (Chart 2)^38^.

Both pegcetacoplan and iptacopan have been shown to be safe and effective and are currently recommended for patients with a glomerular filtration rate above 30 mL/min/1.73 m^2^. All patients who progressed to kidney replacement therapy (dialysis or kidney transplantation) were excluded from the trials. Extension studies with both medications are still ongoing (NCT04817618, NCT05809531). A comparison between the iptacopan and pegcetacoplan trials is shown in Chart 2—important points: (1) more patients in the APPEAR-C3G (iptacopan) trial had nephrotic-range proteinuria, and patients were on a lower dose of steroids compared with the VALIANT (pegcetacoplan) trial; (2) the VALIANT trial also included adolescents (>12 years of age) and kidney transplant recipients.

The recurrence of C3G after kidney transplantation can vary from 60% to 90%; however, there are still no studies on the prophylactic use of anti-complement therapies. This remains a gap in knowledge with the potential to prevent early and late recurrences. In this regard, it is important to note that the VALIANT study included kidney transplant recipients (five patients) who experienced recurrence and responded well to pegcetacoplan. Iptacopan also showed favorable results in 11 kidney transplant patients in a phase 2 trial^39^. Both studies demonstrated safety and efficacy in this patient profile. Pre-transplant genetic testing is recommended, as is testing of related living donors, to enable discussion of recurrence risks with the patient^12^.

Some phase 2 clinical studies with other complement inhibitors have not shown benefit in C3G when compared with placebo, such as avacopan (a C5a inhibitor)^40^ and danicopan^41^. Studies are ongoing to evaluate other complement inhibitors (NCT07156149, NCT06209736).

## Conclusion

C3 glomerulopathy is a biopsy-based diagnosis defined by C3 predominance on immunofluorescence microscopy. EM subdivides C3G into two groups (C3GN and DDD) according to the location and intensity of complement deposits. Recently, ApoE was identified as the main component of osmiophilic deposits in DDD. Both C3GN and DDD are associated with abnormalities of the alternative complement pathway. C3G is a progressive kidney disease with a high rate of recurrence after transplantation. Mycophenolic acid and corticosteroids play a supportive role in controlling the inflammatory process, but they are not specific treatments. Novel anti-complement agents targeting upstream complement regulation have, for the first time, shown promising therapeutic options for C3G. Drugs targeting complement factor B (iptacopan) and complement factors C3/C3b (pegcetacoplan) have reduced proteinuria and attenuated the decline in kidney function in patients with C3G^38,42^. These drugs now provide the option of tailored treatment for selected patients with this disease. Lastly, the availability of both genetic studies and access to serum complement testing in our region remain scarce. Therefore, thorough evaluation of the kidney biopsy is critical to identify a treatable etiology of IC-MPGN and to correctly identify patients with C3G who may benefit from treatment with new anti-complement agents.

## Data Availability

Due to the nature of the review, no data were analyzed.

## References

[1] Smith RJH, Appel GB, Blom AM, Cook HT, D’Agati VD, Fakhouri F (2019). C3 glomerulopathy – understanding a rare complement-driven renal
disease. Nat Rev Nephrol.

[2] Servais A, Noel L-H, Roumenina LT, Le Quintrec M, Ngo S, Dragon-Durey M-A (2012). Acquired and genetic complement abnormalities play a critical
role in dense deposit disease and other C3 glomerulopathies. Kidney Int.

[3] Medjeral-Thomas NR, O’Shaughnessy MM, O’Regan JA, Traynor C, Flanagan M, Wong L (2014). C3 Glomerulopathy: Clinicopathologic Features and Predictors of
Outcome. Clin J Am Soc Nephrol.

[4] Tarragón B, Peleg Y, Jagannathan G, Sekulic M, Chang JH, Cohen DJ (2024). C3 Glomerulopathy recurs early after kidney transplantation in
serial biopsies performed within the first 2 years after
transplantation. Clin J Am Soc Nephrol.

[5] Zand L, Lorenz EC, Cosio FG, Fervenza FC, Nasr SH, Gandhi MJ (2014). Clinical findings, pathology, and outcomes of C3GN after kidney
transplantation. J Am Soc Nephrol.

[6] Burkholder PM, Marchand A, Krueger RP (1970). RP K. Mixed membranous and proliferative glomerulonephritis. A
correlative light, immunofluorescence, and electron microscopic
study. Lab Invest.

[7] Habib R, Gubler MC, Loirat C, Mäiz HB, Levy M (1975). Dense deposit disease: a variant of membranoproliferative
glomerulonephritis. Kidney Int.

[8] Strife CF, Jackson EC, McAdams AJ (1984). Type III membranoproliferative glomerulonephritis: long-term
clinical and morphologic evaluation. Clin Nephrol.

[9] Sethi S, Fervenza FC (2012). Membranoproliferative glomerulonephritis: a new look at an old
entity. N Engl J Med.

[10] Sethi S, Fervenza FC (2011). Membranoproliferative glomeru­lonephritis: pathogenetic
heterogeneity and proposal for a new classification. Semin Nephrol.

[11] Cook HT, Pickering MC (2015). Histopathology of MPGN and C3 glomerulopathies. Nat Rev Nephrol.

[12] Goodship TH, Cook HT, Fakhouri F, Fervenza FC, Frémeaux-Bacchi V, Kavanagh D (2017). Atypical hemolytic uremic syndrome and C3 glomerulopathy:
conclusions from a “Kidney Disease: Improving Global Outcomes” (KDIGO)
Controversies Conference. Kidney Int.

[13] Fakhouri F, Frémeaux-Bacchi V, Noël LH, Cook HT, Pickering MC (2010). C3 glomerulopathy: a new classification. Nat Rev Nephrol.

[14] Fakhouri F, Fremeaux-Bacchi V, Noel L-H, Cook HT, Pickering MC (2010). C3 glomerulopathy: a new classification. Nat Rev Nephrol.

[15] Pickering MC, D’Agati VD, Nester CM, Smith RJ, Haas M, Appel GB (2013). C3 glomerulopathy: consensus report. Kidney Int.

[16] Wooden B, Nester CM, Bomback AS (2024). Update on C3 Glomerulopathy. Adv Kidney Dis Health.

[17] Schena FP, Esposito P, Rossini M (2020). A narrative review on C3 glomerulopathy: a rare renal
disease. Int J Mol Sci.

[18] Smith RJ, Harris CL, Pickering MC (2011). Dense deposit disease. Mol Immunol.

[19] Sethi S, Fervenza FC, Zhang Y, Zand L, Vrana JA, Nasr SH (2012). C3 glomerulonephritis: clinicopathological findings, complement
abnormalities, glomerular proteomic profile, treatment, and
follow-up. Kidney Int.

[20] Ravindran A, Fervenza FC, Smith RJ, Sethi S (2017). C3 glomerulonephritis with a severe crescentic
phenotype. Pediatr Nephrol.

[21] Iatropoulos P, Daina E, Curreri M, Piras R, Valoti E, Mele C (2018). Cluster analysis identifies distinct pathogenetic patterns in C3
glomerulopathies/immune complex-mediated membranoproliferative
GN. J Am Soc Nephrol.

[22] Ravindran A, Pereira Palma LM, Fervenza FC, Sethi S (2023). Overlap of C3 Glomerulopathy and thrombotic micro­angiopathy: a
case series. Kidney Int Rep.

[23] Sethi S, Fervenza FC, Zhang Y, Zand L, Meyer NC, Borsa N (2013). Atypical postinfectious glomerulonephritis is associated with
abnormalities in the alternative pathway of complement. Kidney Int.

[24] Ravindran A, Fervenza FC, Smith RJH, Sethi S (2018). C3 glomerulopathy associated with monoclonal Ig is a distinct
subtype. Kidney Int.

[25] Rovin BH, Adler SG, Barratt J, Bridoux F, Burdge KA, Chan TM (2021). Executive summary of the KDIGO 2021 guideline for the management
of glomerular diseases. Kidney Int.

[26] Caravaca-Fontán F, Polanco N, Villacorta B, Buxeda A, Coca A, Ávila A (2023). Recurrence of immune complex and complement-mediated
membranoproliferative glomerulonephritis in kidney
transplantation. Nephrol Dial Transplant.

[27] Sethi S, Theis JD, Palma LMP, Madden B (2024). From patterns to proteins: mass spectrometry comes of age in
glomerular disease. J Am Soc Nephrol.

[28] Madden B, Singh RD, Haas M, Palma LMP, Sharma A, Vargas MJ (2024). Apolipoprotein E is enriched in dense deposits and is a marker
for dense deposit disease in C3 glomerulopathy. Kidney Int.

[29] Caravaca-Fontán F, Díaz-Encarnación MM, Lucientes L, Cavero T, Cabello V, Ariceta G (2020). Mycophenolate mofetil in C3 glomerulopathy and pathogenic drivers
of the disease. Clin J Am Soc Nephrol.

[30] Ravindran A, Fervenza FC, Smith RJH, De Vriese AS, Sethi S (2018). C3 glomerulopathy: Ten years’ experience at mayo
clinic. Mayo Clin Proc.

[31] Yu BC, Lee MS, Moon JJ, Choi SJ, Kim JK, Hwang SD (2018). Efficacy of low-dose spironolactone on top of angiotensin
receptor blockade in patients with glomerulonephritis. Kidney Res Clin Pract.

[32] Gurkan S, Fyfe B, Weiss L, Xiao X, Zhang Y, Smith RJ (2013). Eculizumab and recurrent C3 glomerulonephritis. Pediatr Nephrol.

[33] Zuber J, Fakhouri F, Roumenina LT, Loirat C, Frémeaux-Bacchi V (2012). Use of eculizumab for atypical haemolytic uraemic syndrome and C3
glomerulopathies. Nat Rev Nephrol.

[34] Wehling C, Amon O, Bommer M, Hoppe B, Kentouche K, Schalk G (2017). Monitoring of complement activation biomarkers and eculizumab in
complement-mediated renal disorders. Clin Exp Immunol.

[35] Bomback AS, Daina E, Remuzzi G, Kanellis J, Kavanagh D, Pickering MC (2025). Efficacy and safety of pegcetacoplan in kidney transplant
recipients with recurrent complement 3 glomerulopathy or primary immune
complex membranoproliferative glomerulonephritis. Kidney Int Rep.

[36] Antonucci L, Thurman JM, Vivarelli M (2024). Complement inhibitors in pediatric kidney diseases: new
therapeutic opportunities. Pediatr Nephrol.

[37] Kavanagh D, Bomback AS, Vivarelli M, Nester CM, Remuzzi G, Zhao MH (2025). Oral iptacopan therapy in patients with C3 glomerulopathy: a
randomised, double-blind, parallel group, multicentre, placebo-controlled,
phase 3 study. Lancet.

[38] Fakhouri F, Bomback AS, Ariceta G, Delmas Y, Dixon BP, Gale DP (2025). Trial of pegcetacoplan in C3 glomerulopathy and immune-complex
MPGN. N Engl J Med.

[39] Wong E, Nester C, Cavero T, Karras A, Le Quintrec M, Lightstone L (2023). Efficacy and safety of iptacopan in patients with C3
glomerulopathy. Kidney Int Rep.

[40] Bomback AS, Herlitz LC, Kedia PP, Petersen J, Yue H, Lafayette RA (2025). Safety and efficacy of avacopan in patients with complement 3
glomerulopathy: randomized, double-blind clinical trial. J Am Soc Nephrol.

[41] Nester C, Appel GB, Bomback AS, Bouman KP, Cook HT, Daina E (2022). Clinical outcomes of patients with C3G or IC-MPGN treated with
the factor D inhibitor danicopan: final results from two phase 2
studies. Am J Nephrol.

[42] Kavanagh D, Bomback AS, Vivarelli M, Nester CM, Remuzzi G, Zhao MH (2025). Oral iptacopan therapy in patients with C3 glomerulopathy: a
randomised, double-blind, parallel group, multicentre, placebo-controlled,
phase 3 study. Lancet.

