# Supplementary material for: C3 Glomerulopathy: recent advances and an update on management
Source: J Bras Nefrol. 2026 Jun 12;48(2):e20250306. doi: 10.1590/2175-8239-JBN-2025-0306en (PMC13263063; doi:10.1590/2175-8239-JBN-2025-0306en)
Supplement: Translation

# Translation: Glomerulopatia C3: avanços recentes e atualizações no manejo

**DOI:** 10.1590/2175-8239-JBN-2025-0306pt

**Authors:** Lilian Monteiro Pereira Palma, Maria Izabel Neves de Holanda Barbosa, Sanjeev Sethi

**Affiliations:** 1 Universidade Estadual de Campinas, Faculdade de Ciências Médicas, Nefrologia Pediátrica, Campinas, SP, Brasil.; 2 Hospital Federal de Bonsucesso, Nefrologia, Rio de Janeiro, RJ, Brasil.; 3 Mayo Clinic, Department of Laboratory Medicine and Pathology, Rochester, MN, United States.

**Keywords:** Glomerulonefrite, Complemento C3, Glomerulonefrite Membranoproliferativa

## Abstract

A glomerulopatia C3 (C3G) é uma entidade clinicopatológica caracterizada por inflamação glomerular com dominância de C3 à imunofluorescência. A microscopia eletrônica é utilizada para determinar a localização e o tipo de depósitos, o que permite subdividir a C3G em glome­rulonefrite C3 (GNC3) e doença de depósitos densos (DDD) com base em achados ultraestruturais. Ambas as entidades são progressivas, com cerca de 60% dos pacientes evoluindo para doença renal terminal (DRT) em 10 anos. A recorrência após o transplante renal ocorre em até 60% dos casos de C3G. Novos conhecimentos sobre a fisiopatologia—diferentes padrões de lesão, escores histopatológicos, coloração para apolipoproteína E, desregulação da via alternativa do complemento, que inclui autoanticorpos e mutações em fatores reguladores do complemento, bem como análises proteômicas—permitiram uma melhor compreensão dos diferentes perfis fisiopatológicos e apresentações clínicas, possibilitando um tratamento individualizado. O papel da nefroproteção está bem estabelecido na C3G, assim como em outras doenças glomerulares. O tratamento não específico com corticosteroides e imunossupressores está associado a uma taxa de resposta variável. No entanto, mais recentemente, os resultados de ensaios clínicos com inibidores proximais da via alternativa do complemento demonstraram melhora significativa na proteinúria e atenuação da redução da taxa de filtração glomerular estimada (TFGe). Com base nos resultados desses ensaios com medicamentos que atuam especificamente nas vias do complemento a montante, incluindo a via alternativa, novas opções de tratamento tornaram-se disponíveis para pacientes com C3G.

## Introdução

A glomerulopatia C3 (C3G) é uma entidade clinicopatológica (isto é, requer biópsia renal para o diagnóstico), geralmente (mas não exclusivamente) caracterizada por um padrão membranoproliferativo (GNMP) de lesão à microscopia de luz, com predominância da coloração de C3 à imunofluorescência. A C3G é ainda subdividida em glomerulonefrite C3 (GNC3) e doença de depósitos densos (DDD) com base na microscopia eletrônica (ME) (Figura 1). Fatores que desregulam a via alternativa do complemento (variantes genéticas e/ou autoanticorpos) estão presentes em uma porcentagem variável de pacientes. Os níveis séricos de C3 estão diminuídos em aproximadamente 60% dos casos. A progressão para doença renal terminal (DRT) em 10 anos é de 70% em crianças e de 30% a 50% em adultos^1^. Além disso, pacientes com DDD apresentam progressão duas vezes mais rápida que pacientes com GNC3^2,3^. Há também uma alta taxa de recorrência após o transplante renal (até 89% em uma série de casos) em uma mediana (intervalo interquartil) de 33 (13–141) dias após o transplante^4^. Embora a sobrevida em curto prazo dos enxertos com recorrência seja favorável, a perda do enxerto em longo prazo pode chegar a 50% em um tempo mediano de 77 meses^5^.

**Figura 1:** Achados de biópsia de glomerulopatia C3 (C3G). A C3G é caracterizada por intensa marcação glomerular para C3 na microscopia de imunofluorescência (IF), na ausência de depósitos significativos de imunoglobulina (Ig). Por definição, a intensidade de C3 é pelo menos duas ordens de magnitude maior do que qualquer Ig. Por outro lado, quando há intensa marcação de Ig na IF, com ou sem C3, a entidade é denominada glomerulonefrite membranoproliferativa mediada por imunocomplexos (GNMP-IC). C3G é subdividida em glomerulonefrite C3 (GNC3) e doença de depósitos densos (DDD) com base na microscopia eletrônica (ME). Na GNC3, os depósitos são subendoteliais, subepiteliais ou intramembranosos esparsos, enquanto, na DDD, os depósitos apresentam formato de salsicha, são densos (osmiofílicos) e intramembranosos. O painel superior mostra os achados da biópsia em um caso de GNC3, e o painel inferior mostra a DDD. Tanto a GNC3 quanto a DDD apresentam um padrão de lesão semelhante à glomerulonefrite membranoproliferativa (GNMP) (A e D), C3 intenso (B e E), com ausência de imunoglobulina (não mostrada), numerosos depósitos subendoteliais na GNC3 (C) e numerosos depósitos densos osmiofílicos intramembranosos na DDD (F).

A glomerulonefrite membranoproliferativa (GNMP) era anteriormente classificada em tipos I, II e III com base na localização dos depósitos eletrodensos por ME^6–8^. Essa classificação não considerava a etiologia subjacente do padrão de lesão da GNMP. Assim, a classificação era problemática, pois causas variáveis do padrão de GNMP eram frequentemente agrupadas em uma única entidade, dificultando o tratamento e a análise dos resultados de ensaios clínicos.

Em 2011, Sethi e Fervenza revisaram a classificação da GNMP, propondo que a microscopia de imunofluorescência fosse a principal ferramenta diagnóstica para diferenciar a GNMP mediada por imunocomplexos/imunoglobulina (Ig) e/ou por deposição de complemento^9,10^. A classificação da GNMP em GNMP mediada por imunocomplexos (GNMP-IC) e GNMP mediada por complemento alterou a abordagem fundamental, passando de uma base morfológica para uma base etiológica do padrão de GNMP^11,12^. Assim, a GNMP mediada por imunocomplexos/Ig está mais associada a infecções, doenças autoimunes ou paraproteinemia, enquanto a GNMP mediada pelo complemento está associada a anormalidades da via alternativa do complemento (Quadro 1).

**Quadro 1:** Investigação de pacientes com glomerulonefrite C3 (GNC3) e glomerulonefrite membranoproliferativa mediada por imunocomplexos (GNMP-IC)

Descartar causas secundárias e avaliar defeitos na via alternativa do complemento
Causas secundárias: • Hepatite B • Hepatite C • Vírus da Imunodeficiência Humana • Pesquisa de protozoários • Doenças tropicais: malária, esquistossomose • Doenças autoimunes • Gamopatia monoclonal (em todas as idades, especialmente em pacientes com idade > 50 anos) • Doença falciforme • Infecções agudas, endocardite • Infecção estreptocócica • Neoplasias
Quantificação de fatores do complemento e reguladores: • C3 • C4 • CH50 • AH50 • Fator H, Fator I, Fator B • Anticorpo anti-fator H • C3 nefritogênico (C3NeF) • C4 nefritogênico (C4NeF) • C5 nefritogênico (C5NeF)
Painel genético ou sequenciamento completo do EXOMA

Ao mesmo tempo, o termo C3G foi proposto em 2010 por um grupo francês após analisar biópsias que mostravam glomerulonefrite com coloração dominante de C3 na microscopia de imunofluorescência, alertando para a possibilidade de defeitos na via alternativa do complemento^13,14^. Esses estudos levaram a uma reunião de consenso em 2013, a fim de obter uma definição uniforme de glomerulonefrite mediada pelo complemento. Reconheceu-se que, embora a GNMP fosse o padrão de lesão mais comum, outros padrões de lesão, incluindo glomerulonefrite proliferativa mesangial, glomerulonefrite crescente e glomeru­lonefrite proliferativa endocapilar difusa, também podem estar presentes nesse grupo de doenças^15^. A terminologia GNMP mediada pelo complemento foi, portanto, substituída pela terminologia C3G.

A importância da classificação correta da GNMP em GNMP-IC e C3G não pode ser subestimada. A avaliação clínica, patológica e laboratorial detalhada da GNMP-IC leva, muitas vezes, à identificação da etiologia subjacente, que então se torna passível de tratamento direcionado (Sethi S, *Kidney Int*, 2026, revisão menor submetida). O termo GNMP primária/idiopática é utilizado quando uma etiologia subjacente dos imunocomplexos/Ig não é encontrada. Por outro lado, o diagnóstico de C3G deve levar à avaliação de anormalidades do complemento, especialmente na via alternativa. Esses pacientes provavelmente se beneficiarão mais de medicamentos que bloqueiam as vias do complemento. Como a GNMP-IC idiopática também apresenta sinais de ativação do complemento e deposição glomerular desses fatores, é fácil imaginar que o bloqueio desse sistema igualmente tenha um efeito benéfico nesse grupo de pacientes.

## Discussão

A C3G é uma doença glomerular ultrarrara, com incidência de 1 a 2 novos casos por milhão de habitantes por ano, cujas manifestações clínicas mais frequentes são hematúria e proteinúria, podendo apresentar-se como síndrome nefrítica e/ou nefrótica, em alguns casos, com baixos níveis de C3^16,17^. A Sociedade Brasileira de Nefrologia lançou o Registro C3G/GNMP-IC por meio de seu Comitê de Doenças Raras (comdora-sbn.org.br—acesso restrito), visto que não há dados sobre a prevalência dessa doença no país ou na região. Além do envolvimento renal, pacientes com DDD podem apresentar manifestações extrarrenais, como drusas oculares e lipodistrofia adquirida^18^.

A base fisiopatológica da C3G é uma desregulação da via alternativa do complemento que pode ocorrer devido a mutações em proteínas reguladoras do complemento (menos de 20% dos casos) ou devido a causas adquiridas, que incluem os fatores nefrotóxicos C3, C4 e C5 (C3NeF, C4NeF, C5NeF—autoanticorpos que estabilizam a convertase C3/C5 da via alternativa) e autoanticorpos anti-fator B, anti-fator I e anti-fator H^19^. Todos esses mecanismos levam à ativação excessiva do complemento, com consequente acúmulo glomerular de fatores do complemento. Como esses fatores são potentes agentes quimiotáticos, ocorre um influxo de leucócitos (fase proliferativa), seguido pela formação de uma nova membrana basal (fase de reparo), o que leva a uma aparência de dupla membrana à microscopia de luz, a qual é a característica principal do padrão de lesão da GNMP. Outros padrões de lesão, além da GNMP, podem estar presentes, incluindo os padrões proliferativo mesangial, crescêntico e esclerosante^20,21^. Iatropoulos et al. avaliaram 173 pacientes com C3G e GNMP-IC, separando-os em quatro grupos de acordo com critérios de biópsia, marcadores de complemento sérico e achados genéticos, sugerindo variabilidade na resposta ao tratamento e no desfecho entre os diferentes grupos^21^. A sobreposição de C3G e síndrome hemolítica urêmica atípica (SHUa) foi descrita em pacientes que são mais propensos a apresentar variantes genéticas em genes da via alternativa do complemento, em vez de fatores nefríticos^22^.

A maioria dos casos de glomerulonefrite pós-infecciosa (GNPI) apresenta um padrão exsudativo de lesão, com IgG granular e C3 intenso ao longo das paredes capilares na microscopia de imunofluorescência. No entanto, não é incomum que a GNPI apresente deposição intensa de C3, com escassa imunoglobulina na microscopia de imunofluorescência. Nesses casos, a distinção entre GNPI e GNC3 dependerá da ausência de características atípicas na microscopia de luz e eletrônica, bem como de um curso clínico típico de GNPI, com resolução completa e normalização do C3 sérico em até oito semanas. Contudo, é importante notar que a GNC3 pode se manifestar após um episódio infeccioso, e pode haver protuberâncias subepiteliais (*humps*) tanto na GNC3 quanto na GNPI^23^. Portanto, a presença de quaisquer características clínicas ou histológicas atípicas em um caso de GNPI deve levantar a suspeita de GNC3.

Outro diagnóstico diferencial importante é a presença de uma imunoglobulina monoclonal, a qual foi relatada em casos de GNC3 sem depósitos clonais no tecido renal^24^. A Ig monoclonal provavelmente atua como um autoanticorpo contra proteínas reguladoras do complemento, resultando em desregulação da via alternativa. Portanto, recomenda-se que a paraproteinemia seja investigada em todos os pacientes com GNC3 (especialmente aqueles com mais de 50 anos). Recomenda-se também a consulta com um hematologista, pois o tratamento da paraproteinemia subjacente, com terapia direcionada ao clone, frequentemente leva à remissão da doença renal em casos de GNC3 associada a Ig monoclonal^25^. Deve-se ressaltar que há uma elevada taxa de recorrência após o transplante nesses casos^26^. Assim, a identificação desse subgrupo de pacientes torna-se ainda mais importante.

Mais recentemente, a microdissecção a laser e a espectrometria de massa (LMD/MS) em doenças glomerulares contribuíram para a compreensão dos mecanismos fisiopatológicos da C3G. A descoberta mais relevante decorre de um estudo liderado pela Clínica Mayo, em Rochester, no qual a LMD/MS mostrou acúmulo acentuado de proteínas do complemento e de proteínas reguladoras do complemento tanto na GNC3 quanto na DDD, em comparação com os controles^27,28^. Um aumento de seis a nove vezes no C5-9 (complemento terminal) foi detectado em casos de DDD em comparação com a GNC3. Além disso, um aumento de nove vezes na quantidade de apolipoproteína E (ApoE) foi surpreendentemente encontrado em biópsias de DDD em comparação com a GNC3. Estudos de validação confirmaram o diagnóstico de GNC3 e DDD em 80,6% dos casos, com base apenas na coloração de ApoE. Este marcador pode ser utilizado como complemento à ME para o diagnóstico de DDD, podendo ser valioso quando a ME não estiver disponível^28^.

Publicada no *Kidney International* em 2021, a Diretriz de Prática Clínica para Doenças Glomerulares da *Kidney Disease: Improving Global Outcomes* (KDIGO)^25^ estratifica o manejo da C3G e da GNMP-IC. O primeiro passo (Figura 2) é tratar a condição subjacente sempre que identificada (infecção, disproteinemia, doença autoimune e outras). O segundo passo é tratar os pacientes de acordo com a função renal e o grau de proteinúria. Para pacientes com C3G, função renal normal e proteinúria em níveis nefróticos ou sedimento urinário ativo, corticosteroides podem ser utilizados por 12 a 16 semanas (prednisona 1 mg/kg/dia ou equivalente), em conjunto com medidas nefroprotetoras tradicionais (inibidores da enzima conversora de angiotensina, bloqueadores dos receptores de angiotensina em doses máximas toleradas, dieta, controle lipídico). À medida que a filtração glomerular diminui ou a proteinúria persiste, o uso de imunossupressores (especialmente o ácido micofenólico) tem sido indicado em casos moderados da doença (proteinúria acima de 500 mg/dia, inflamação moderada na biópsia ou aumento agudo da creatinina sérica)^29^. Na população europeia, a remissão com ácido micofenólico foi alcançada principalmente em pacientes jovens com C3NeF e autoanticorpos. Houve recorrência da proteinúria em 33% dos pacientes que interromperam o uso de ácido micofenólico. É importante, no entanto, destacar que seu uso nesse contexto ainda é considerado *off-label*, e esses resultados não foram observados em uma série de casos dos Estados Unidos^30^. Em doenças graves com proteinúria superior a 2 g/dia (ou inflamação grave na biópsia ou perda progressiva da função renal), apesar da imunossupressão e da terapia de suporte, pode-se utilizar terapia de pulso com metilprednisolona ou medicamentos anticelulares, embora a eficácia não tenha sido comprovada.

**Figura 2:** Uma nova proposta de manejo de pacientes com glomerulopatia C3.

Nos casos em que se observa uma forma crescente na biópsia, indica-se a combinação de ciclofosfamida e corticosteroides em altas doses^25^.

O uso de inibidores do cotransportador sódio-glicose 2 (SGLT2i) e de antagonistas do receptor de mineralocorticoides tem sido associado ao aumento da sobrevida do paciente e da sobrevida renal, mesmo em pacientes não diabéticos com filtração glomerular estimada entre 25 e 75 mL/min/1,73 m^2^. Essas terapias estão emergindo como tratamento não específico que pode ser utilizado em pacientes com GNC3 e proteinúria^25,31^.

Durante vários anos, o único inibidor do complemento disponível no mercado foi o eculizumabe, um anticorpo monoclonal anti-C5 humanizado. O uso *off-label* para alguns casos de C3G não se mostrou eficaz em todos os pacientes^32,33^, incluindo a coorte europeia composta por 97 pacientes com C3G, dos quais nove receberam eculizumabe sem benefício para a sobrevida renal^29^. Isso provavelmente se deve ao seu efeito a jusante, enquanto o defeito do complemento na C3G ocorre principalmente a montante. Esse achado foi demonstrado em um estudo que avaliou biomarcadores em pacientes com SHUa, C3G e rejeição aguda mediada por anticorpos, tratados com eculizumabe, no qual indivíduos com C3G mantiveram níveis elevados de C3d e níveis consistentemente baixos de C3 com inibição terminal do complemento, refletindo ativação proximal contínua, apesar do uso de um inibidor terminal^34^.

Recentemente, novos medicamentos foram aprovados pelo *Food and Drug Administration* (FDA) para o tratamento de C3G^35,36^ (Quadro 2).

**Quadro 2:** Ensaios clínicos com inibidores de C3 – dados demográficos e resultados

Estudo Clínico	APPEAR (NCT04817618) – Novartis Pharma	VALIANT (NCT05067127) – Apellis Pharma
Droga	Iptacopan (Inibidor do Fator B)	Pegcetacoplan (Inibidor de C3/C3b)
Administração	200 mg via oral, a cada 12 horas	1080 mg por via subcutânea, 2 vezes por semana
Doença	Rins nativos, C3G (biópsia confirmada em até 12 meses após a inclusão)	C3G (n = 51), GNMP-IC (n = 12) em rins nativos e pós-transplante
Desenho do Estudo	Estudo multicêntrico, randomizado, duplo-cego, controlado por placebo, de fase 3	Estudo multicêntrico, randomizado, duplo-cego, controlado por placebo, de fase 3
Critérios de Inclusão	*Adultos de 18 a 30 anos *Concentração sérica reduzida de C3 ( < 77 mg/dL [definida como < 0,85 × limite inferior da faixa normal do laboratório central]) na triagem *Uso de MPA, corticosteroides (prednisona < 7,5 mg/dia), iSGLT2 e ARM permitido quando estável por pelo menos 90 dias antes da randomização *Dose máxima tolerada de inibidores da enzima conversora de angiotensina ou bloqueadores do receptor AT1 por pelo menos 90 dias *RPCu ≥1,0 g/g no dia -75 e no dia -15 antes da randomização *TFGe ≥ 30 mL/min/1,73 m^2^ *Vacinação contra *Neisseria meningitidis* e *Streptococcus pneumoniae*	*Adolescentes (12–17 anos) e adultos (≥18 anos) *Rins nativos e recorrência pós-transplante renal *Uso de MPA e corticosteroides (prednisona ≤ 20 mg/dia) permitido *Regimes antiproteinúricos estáveis e otimizados: iECA, BRA, iSGLT2 *RPCu ≥1,0 g/g em 2 ou mais amostras de urina da primeira micção da manhã e durante a triagem *TFGe ≥30 mL/min/1,73 m^2^ *Vacinação contra *Streptococcus pneumoniae*, *Neisseria meningitidis* e *Haemophilus influenzae* tipo B
Critérios de Exclusão	*Receptor de qualquer transplante de células ou órgão sólido (para fase 3) *Glomerulonefrite crescente rapidamente progressiva *Biópsia renal exibindo fibrose intersticial ou atrofia tubular *Gamopatia monoclonal de significado indeterminado	*Evidência de rejeição do transplante *Diagnóstico de C3G secundária ou GNMP-IC *Infecção grave nos 14 dias anteriores à primeira dose; infecções graves recorrentes ou crônicas ou histórico de doença meningocócica *Evidência de melhora da doença renal
Desfecho Primário	Redução da proteinúria (medida pela razão logarítmica em relação ao valor basal da RPCu em amostra de urina coletada em 24 horas) aos 6 meses.	Razão logarítmica da RPCu na semana 26, em comparação com o valor basal.
Participantes (n)	Grupo iptacopan (38)/grupo placebo (36)	Grupo pegcetacoplan (63)—C3G (51), GNMP-IC (12); adultos (35); adolescentes (28); rim nativo (58), transplante renal (5) Grupo placebo (61)—C3G (45), GNMP-IC (16); adultos (34); adolescentes (27); rim nativo (57), transplante renal (4)
Pós-transplante renal	Analisado em estudo de fase 2 (11 C3G)	Grupo pegcetacoplan (5) /grupo placebo (4)
Histopatologia	Alteração média na pontuação total de atividade do início do estudo até 6 meses: -1,95 (iptacopan) vs. 1,11 (placebo) (p = 0,2895) Alteração média na pontuação de depósito de C3 do início do estudo até 6 meses: -0,78 (iptacopan) vs. 1,09 (placebo)	Alteração média na pontuação total de atividade do início do estudo até 6 meses: -3,5 (pegcetacoplan) vs. -2,4 (placebo) (p = 0,2753) Redução de mais de duas ordens de magnitude na coloração de C3c: 74,3% (pegcetacoplan) vs. 11,8% (placebo)
RPCu mudança vs. placebo	35,1%	68,3%
Desfecho renal composto (≥50% de redução na RPCu E ≤15% de redução na TFGe): semana 26 vs. basal	Aumento de 7,15 vezes (IC 95% 1,43–35,72) na probabilidade de atingir o desfecho renal composto, em comparação com o placebo (p = 0,0166) Aumento da TFGe em 6 meses: +1,30 mL/min/1,73 m^2^ (iptacopan) vs. -0,86 mL/min/1,73 m^2^ (placebo)	Probabilidade 29,4 vezes maior (IC 95% 6,5–132,2) de atingir os desfechos renais compostos com pegcetacoplan, em comparação com o placebo (p < 0,0001)
Pacientes com proteinúria basal > 3g/24h (%)	Grupo iptacopan (71%)/grupo placebo (58%)	Grupo pegcetacoplan (38%)/grupo placebo (23%)
TFGe basal (média)	Grupo iptacopan (89,3 mL/min/1,73 m^2^)/grupo placebo (99,2 mL/min/1,73 m^2^)	Alteração média da TFGe em relação ao valor basal: -1,6 mL/min/1,73 m^2^ (pegcetacoplan) vs. -7,9 mL/min/1,73 m^2^ (placebo)
Eventos Adversos Graves	Grupo iptacopan (8%)/grupo placebo (3%)	Grupo pegcetacoplan (9,5%) – 1 óbito devido à COVID-19/grupo placebo (9,8%)

Abreviaturas – RPCu: relação proteína/creatinina urinária; TFGe: taxa de filtração glomerular estimada; C3G: glomerulopatia C3; GNMP-IC: glomerulonefrite membranoproliferativa por imunocomplexos; iECA: inibidores da enzima conversora de angiotensina; BRA: bloqueadores do receptor da angiotensina; GNC3: glomerulonefrite C3; DDD: doença de depósito denso; MPA: ácido micofenólico; iSGLT2: inibidores do cotransportador sódio-glicose 2; ARM: antagonistas do receptor de mineralocorticoides; IC: intervalo de confiança.

O estudo APPEAR-C3G (Novartis Pharma) foi um ensaio de fase 3, prospectivo, multicêntrico, randomizado, duplo-cego e controlado por placebo, que avaliou o uso de iptacopan em pacientes com C3G—uma molécula da classe dos indóis e um potente inibidor do fator B do complemento, que previne a amplificação desregulada da C3 convertase. Ambos os braços receberam cuidados de suporte. O iptacopan foi administrado na dose de 200 mg por via oral, duas vezes ao dia. Os desfechos incluíram redução da proteinúria, medida pela relação proteína/creatinina urinária (RPCU), e pela inclinação da taxa de filtração glomerular estimada (TFGe). Nos pacientes que completaram seis meses de terapia, o iptacopan reduziu a proteinúria em 35,1% em comparação com o placebo^37^, e 43,5% dos pacientes atingiram o desfecho composto (redução >50% na RPCu + redução < 15% na TFGe) em 12 meses.

O estudo VALIANT (Apellis Pharma), um estudo prospectivo, randomizado, duplo-cego de fase 3, demonstrou que o uso de pegcetacoplan—um peptídeo peguilado que se liga seletivamente ao C3 e impede sua clivagem em C3a e C3b—em pacientes com C3G e GNMP-IC, em comparação com o placebo por 26 semanas, apresentou redução de 68,3% na proteinúria dos pacientes tratados. O pegcetacoplan foi administrado por via subcutânea, na dose de 1080 mg duas vezes por semana. A proporção de pacientes que atingiram o desfecho renal composto (redução ≥50% na RPCU, associada à redução ≤15% na TFGe) na semana 26, em comparação com o valor basal, foi de 49,2% no grupo pegcetacoplan, com probabilidade 29,4 vezes maior de atingir os desfechos renais compostos em comparação com o placebo (p < 0,0001). No grupo pegcetacoplan, houve um ganho de 6,3 mL/min/1,73 m^2^ na TFGe, quando comparado ao do placebo (Quadro 2)^38^.

Tanto o pegcetacoplan quanto o iptacopan demonstraram ser seguros e eficazes e são atualmente recomendados para pacientes com taxa de filtração glomerular acima de 30 mL/min/1,73 m^2^. Todos os pacientes que progrediram para terapia de substituição renal (diálise ou transplante renal) foram excluídos dos ensaios. Estudos de extensão com ambos os medicamentos ainda estão em andamento (NCT04817618, NCT05809531). Uma comparação entre os ensaios com iptacopan e pegcetacoplan é demonstrada no Quadro 2—pontos importantes: (1) mais pacientes no ensaio APPEAR-C3G (iptacopan) apresentaram proteinúria em níveis nefróticos, e os pacientes estavam em uma dose menor de esteroides em comparação com o ensaio VALIANT (pegcetacoplan); (2) o ensaio VALIANT também incluiu adolescentes (> 12 anos de idade) e receptores de transplante renal.

A recorrência da C3G em pacientes transplantados renais pode variar de 60% a 90%; porém, ainda não existem estudos sobre o uso profilático de medicamentos anti-complemento. Essa lacuna no conhecimento apresenta potencial para prevenir recorrências precoces e tardias. Nesse sentido, é importante destacar que o estudo VALIANT incluiu receptores de transplante renal (cinco pacientes) que apresentaram recorrência e responderam bem ao pegcetacoplan; além disso, o iptacopan apresentou resultados favoráveis em 11 pacientes transplantados renais em um ensaio de fase 2^39^. Ambos os estudos demonstraram segurança e eficácia nesse perfil de pacientes. Recomenda-se o teste genético pré-transplante renal, assim como o teste de doadores vivos relacionados, de modo a permitir a discussão dos riscos de recorrência com o paciente^12^.

Alguns estudos clínicos de fase 2 com outros inibidores do complemento não mostraram benefício para C3G quando comparados ao placebo, como avacopan (inibidor de C5a)^40^ e danicopan^41^. Estudos estão em andamento para avaliar outros inibidores do complemento (NCT07156149, NCT06209736).

## Conclusão

A glomerulopatia C3 é um diagnóstico por biópsia, baseado na predominância de C3 na microscopia de imunofluorescência. A ME subdivide a C3G em dois grupos (GNC3 e DDD), de acordo com a localização e a intensidade dos depósitos de complemento. Recentemente, a ApoE foi identificada como o principal componente dos depósitos osmiofílicos na DDD. Tanto a GNC3 quanto a DDD estão associadas a anormalidades na via alternativa do complemento. A C3G é uma doença renal progressiva, com elevada taxa de recorrência em pacientes transplantados. O ácido micofenólico e os corticosteroides desempenham um papel de suporte no controle do processo inflamatório, mas não são tratamentos específicos. Novos agentes anti-complemento, que têm como alvo a regulação a montante das vias do complemento, demonstraram, pela primeira vez, opções promissoras para o tratamento da C3G. Medicamentos que têm como alvo o fator B (iptacopan) e os fatores C3/C3b do complemento (pegcetacoplan) diminuíram a proteinúria e atenuaram a deterioração da função renal em pacientes com C3G^38,42^. Esses medicamentos agora oferecem a opção de tratamento personalizado para pacientes selecionados acometidos por essa condição. Por fim, a disponibilidade de estudos genéticos e o acesso a estudos do complemento sérico em nossa região ainda são escassos. Portanto, a avaliação minuciosa da biópsia renal é extremamente importante para identificar uma etiologia tratável de GNMP-IC e para identificar corretamente os pacientes com C3G que podem se beneficiar do tratamento com novos agentes anti-complemento.

## Disponibilidade de Dados

Devido à natureza da revisão, não há dados disponíveis para análise.
